# A comparative analysis of SARS-CoV-2 viral load across different altitudes

**DOI:** 10.1038/s41598-022-20516-w

**Published:** 2022-10-13

**Authors:** Esteban Ortiz-Prado, Raul Fernandez-Naranjo, Jorge Eduardo Vásconez, Alexander Paolo Vallejo-Janeta, Diana Morales-Jadan, Ismar A. Rivera-Olivero, Tannya Lozada, Gines Viscor, Miguel Angel Garcia-Bereguiain, The UDLA-COVID-19 team, Jonathan Dario Rondal, Genoveva Granda, Ana Cecilia Santamaria, Cynthia Lorena Pino, Oscar Lenin Espinosa, Angie Buitron, David Sanchez Grisales, Karina Beatriz Jimenez, Vanessa Bastidas, Dayana Marcela Aguilar, Ines Maria Paredes, Christian David Bilvao, Sebastian Rodriguez Pazmiño, Juan Carlos Laglaguano, Henry Herrera, Pablo Marcelo Espinosa, Edison Andres Galarraga, Marlon Steven Zambrano-Mila, Ana Maria Tito, Nelson David Zapata

**Affiliations:** 1 One Health Research Group, Faculty of Medicine, Universidad de Las Americas, Calle de los Colimes y Avenida De los Granados, 17013 Quito, Ecuador; 2 Department of Cell Biology, Physiology, and Immunology of the Faculty of Biology, Universitat de Barcelona, Barcelona, Spain; 3 Facultad de Ciencias de la Salud, Universidad Latina de Costa Rica, San Pedro, Costa Rica; 4 Decanato de Investigación y Vinculación, Universidad de Las Americas, Quito, Ecuador

**Keywords:** Climate sciences, Diseases, Health occupations

## Abstract

SARS-CoV-2 has spread throughout the world, including areas located at high or very high altitudes. There is a debate about the role of high altitude hypoxia on viral transmission, incidence, and COVID-19 related mortality. This is the first comparison of SARS-CoV-2 viral load across elevations ranging from 0 to 4300 m. To describe the SARS-CoV-2 viral load across samples coming from 62 cities located at low, moderate, high, and very high altitudes in Ecuador. An observational analysis of viral loads among nasopharyngeal swap samples coming from a cohort of 4929 patients with a RT-qPCR test positive for SARS-CoV-2. The relationship between high and low altitude only considering our sample of 4929 persons is equal in both cases and not significative (*p*-value 0.19). In the case of low altitude, adding the sex variable to the analysis, it was possible to find a significative difference between men and women (*p*-value < 0.05). Considering initially sex and then altitude, it was possible to find a significative difference between high and low altitude for men (*p*-value 0.05). There is not enough evidence to state that viral load is affected directly by altitude range but adding a new variable as sex in the analysis shows that the presence of new variables influences the relationship of altitude range and viral load. There is no evidence that viral loads (Ct and copies/ml) differ at low or high altitude. Using sex as a co-factor, we found that men have higher viral loads than women at low and moderate altitude locations, while living at high altitude, no differences were found. When Ct values were aggregated by low, moderate, and high viral load, we found no significant differences when sex was excluded from the analysis. We conclude that viral load is not directly affected by altitude, but COVID-19 incidence and mortality are rather affected by socio-demographic and idiosyncratic dynamics.

## Introduction

The COVID-19 pandemic has been one of the most serious public health problems of the last century^1^. Since its declaration, more than 600 million confirmed cases had been reported worldwide^2^. While the number of deaths has not yet exceeded 7 million, unofficially, excess deaths have been estimated at more than 20 million worldwide^3,4^. Ecuador as well as most of the countries in Latin America, were the most affected, especially in the early stages of the outbreak^5^. For example, Ecuador suffered the most lethal COVID-19 related wave of the pandemic in the first trimester of 2020, reporting more than 700% more deaths per capita than the United Sated of the America on its most critical day^6^. For example, the USA reported just over 5000 deaths in a single day (1.5 deaths/100,000), Brazil reported 4,300 deaths in a single day (1.9/100,000), and Peru reported just over 700 deaths in a single day (2.4/100,000), but Ecuador reported over 1200 deaths in a single day (6.7/100,000)^3^.

Since then, certain interrogations remain unsolved concerning viral transmission and the burden of COVID-19 at different altitudes^7–12^. The role of high altitude exposure on COVID-19 attack rate or SARS-CoV-2 infection has generated controversy and intrigued the scientific community^13^, nevertheless, there are still various questions surrounding this alleged relationship. One of the reasons behind these unresolved doubts might be due to the fact that most of the countries worldwide have their populations living at low altitudes, while some of the countries that are actually located at high altitude, have no low-altitude towns to compare with, except some Andean and very few Asian countries’^14^. In this sense, classifying high altitude populations is not an easy task. Often, the arbitrary low (< 2500 m) and high (> 2500 m) altitude range have been used. Nevertheless, Imray et al., 2011 proposed a better suited classification of high-altitude exposure based in Pollard & Murdoch work published in “The High Altitude Medicine Handbook". They used a categorization that seems to be the most pragmatic and has been widely adopted by the mountain medicine community^15^. According to this categorization, low altitude is defined as everything located below 1500 m, moderate or intermediate altitude between 1500 m to 2500 m, high-altitude from 2500 m to 3500 m, the very high-altitude from 3500 m to 5800 m, more than 5800 m extreme high-altitude and finally above the 8000 m is considered the death zone^15^.

Since the very beginning of the pandemic, some researchers have proposed that hypobaric hypoxia could act as a protective factor against SARS-CoV-2 attack rate or COVID-19 related mortality^9,13,16,17^. This proposed effect might be attributed to several factors such as the prevalence of chronic diseases, a mostly aging population, socioeconomic differences and inequalities in terms of access to medical services^18^; However, the role of different environmental factors such as humidity, temperature or low barometric pressures on the transmission of SARS-CoV-2 at high altitude have also been proposed^18,19^.

The hypotheses surrounding the role of high altitude on SARS-CoV-2 transmission and the impact of the pandemic in these populations can be classified in three main groups: (1) The physiological and biological role of altitude-adapted organisms in relation to virus transmission or replication (i.e. the role of ACE-2 receptors at high altitude), (2) the epidemiological relationship between sociodemographic factors and COVID-19 incidence and mortality at high altitude (i.e. population density, overcrowding activities or migration) and lastly, (3) the direct or indirect consequences of the environment on virulence or viral transmission (i.e. ozone, UV exposure or cold)^8,9,12,13,17,20^.

From these, the least studied factor has been the possible influence of living at high altitude where hypobaric hypoxia could influence viral transmission or viral replication. In this sense, a suitable indicator would be the evaluation of viral load using the reverse transcription polymerase chain reaction (RT-PCR) test across patients with a positive diagnosis of SARS-CoV-2 infection^21,22^. Using the number of cycles (Ct) required to detect the presence of the SARS-CoV-2 within a RT-qPCR test have been widely used^23^.

The aim of this study was to compare SARS-CoV-2 viral load across patients living in different jurisdictions located at low, moderate, high or very high altitudes in Ecuador.

## Methods and data

### Study design

An observational, ecological analysis of 4929 patients that tested positive for SARS-CoV-2 infection as part of our COVID-19 detection-campaign across 62 jurisdictions in Ecuador during May 2020 to October 2020 was carried out.

### Setting

**Figure 1:**
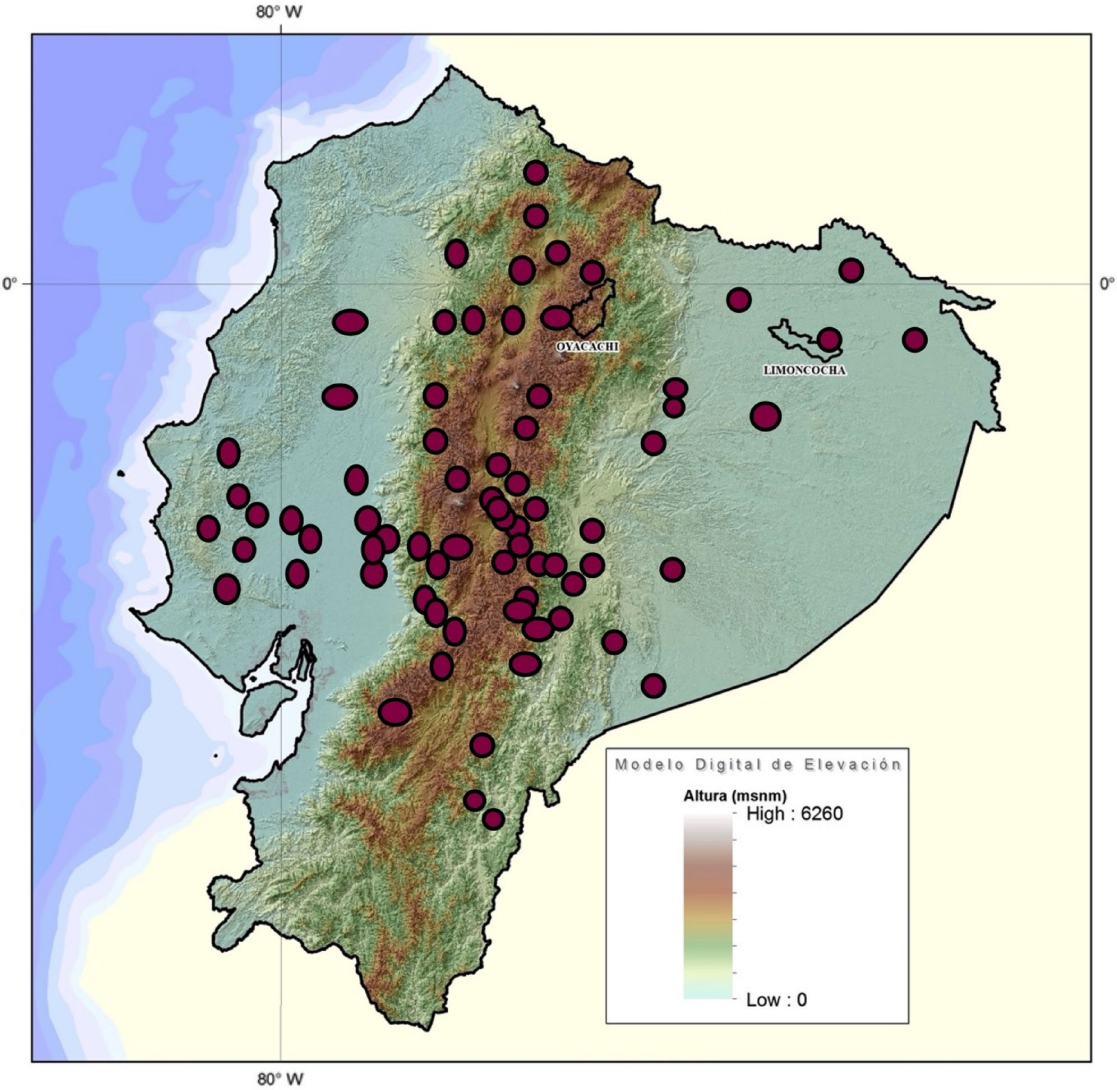
Topographic map of Ecuador including elevation scale (Map created by the authors using ArcGIS Version 10.3).

The study was carried out in Ecuador, one of the smallest Latin-American countries, located in the equatorial line and bordering the Pacific Ocean. Ecuador shares borders with Peru and Colombia and its current population is estimated to be 17,577,116 inhabitants. The country has four regions (Coastal Lowlands, The Andean Highlands, The Amazonian Basin, and the Galapagos Islands) organized in 24 provinces and 221 political subdivisions called cantons (cities) with an elevation range from 0 to 4300 m (Fig. 1). The population density in Ecuador is 71 per km^2^ (184 people per mi^2^), the total land area is 248,360 km^2^ (95,892 mi^2^), 63% of the population is urban (11,123,641 people in 2020) and the median age in Ecuador is 27.9 years^2^.

### Population

The sampling included all persons who voluntarily approached our diagnostic station to have their SARS-CoV-2 viral status during our national SARS-CoV-2 detection countrywide campaign. More than 22,000 subjects were sampled countrywide and from those, 4929 patients tested positive for SARS-CoV-2 infection during this time.

### Data source and variables

After obtaining the informed consent, an epidemiological data-recollection sheet form was completed, including demographic variables including sex, age, jurisdiction of residence as well as symptomology status were obtained. For nasopharyngeal swabs samples, the Center for Disease Control (CDC) 2019-Novel Coronavirus (2019-nCoV) RT-qPCR Diagnostic Panel was used to identify the presence of SARS-CoV-2. All samples were processed in the BSL2 certified molecular biology laboratory at Universidad de Las Americas.

### RNA Extraction and RT-qPCR for SARS-CoV-2 diagnosis using 2019-nCoV CDC kit

The samples were processed in the BSL2 certified molecular biology laboratory at Universidad de Las Americas. Nasopharyngeal swabs were collected on 0.5 mL TE pH 8 buffer for SARS-CoV-2 diagnosis by RT-qPCR following an adapted version of the CDC protocol as it has been previously described by our laboratory. Briefly, the CDC RT-qPCR protocol is based on N1 and N2 gene targets to detect SARS-CoV-2 and RNase P gene target as an RNA extraction quality control Also, negative controls (TE pH 8 buffer) were included as control for carryover contamination, one for each set of RNA extractions, to guarantee that only true positives were reported^24–27^. The criteria for SARS-CoV-2 positivity were a dual amplification of N1 and N2 with Ct values below 40, or a single amplification of either N1 or N2 with Ct values below 40 by duplicate^24–27^. For viral loads calculation, Ct values for N1 viral target were used and referred to a calibration curve done with the 2019-nCoV N positive control (IDT, USA), provided at 200.000 genome equivalents/µL; a factor of 200 was applied to convert the viral loads to genome equivalents/mL and then converted to logarithmic scale^24–27^.

### Variables

Data from 4929 samples that tested positive for SARS-CoV-2 infection were included. The information from every patient was categorized by Sex (Female or Male), age (0–100 years), viral load (copies/ml), cycle threshold (Ct) and altitude ranges (0–4300 m). Since several investigations use 2500 m as the elevation threshold to define high altitude, we classified our data as follow: Low altitude (anything below 2500 m) and high altitude (anything above 2500 m). Due to the wide range of altitudes found in Ecuador, we also used the International Society of Mountain Medicine classification as: Low altitude (0–1500 m), moderate altitude (1500–2500 m), high altitude (2500–3500 m) and very high altitude (3500 to 5800 m).

### Study and sample Size

From the SARS-CoV-2 detection program, we included only the samples that resulted positive. A total of 4,929 subjects from 62 jurisdictions in Ecuador were analyzed. We used a non-probabilistic convenience sample technique until the desire number of positive samples was achieved. From our data, 43% (*n* = 2135) came from positive samples from those living in low altitude jurisdictions and 57% (*n* = 2794) came from those living at high altitude.

### Statistical methods

Descriptive and inferential analysis was conducted using the software IBM SPSS Statistics for Windows Version 24.0. Figures and graphs were made using the official R software, an open-source free software environment. Measurements of frequency (counts, absolute and relative percentages), central tendency (median), dispersion (interquartile range) and absolute differences were calculated for all categorical and continuous variables when justified.

A t-test analysis parametric or a Wilcoxon–Mann–Whitney nonparametric tests were used to asses’ differences when indicated.

The hypotheses used can be explained as:

#### H_0_

Viral load at low and high altitude are equal.

#### H1

Viral load at low and high altitude are different.

Having a wide range of elevations and other covariates as sex, we also tested the hypothesis:

#### H0

Viral load is equal across all altitude ranges.

#### H1

Viral load depends on other variables.

### Ethical approval

All participants signed an informed consent to participate freely and voluntarily in this SARS-CoV-2 testing surveillance program. This study is a secondary analysis of the anonymized laboratory results from a previous surveillance testing done in the context of COVID-19 pandemic. Nevertheless, the study was approved by Institutional Review Board from Hospital General San Francisco (Quito) with code CEISH-HGSF-2021-002. All procedures performed in our study were in accordance with the ethical standards of the Minister of Public Health (MoH) and with the Helsinki Declaration and comparable ethical standards.

### Bias

Sample collection and data analysis were performed by qualified personnel from the Universidad de las Americas in the presence of MoH staff. The analysis and interpretation of the data was done with 2 of the investigators separately to look for discrepancies. Any new findings were reviewed by the entire team and a unanimous decision was made in the event of differences in the results. Despite having 4929 observations in our sample and achieving the necessary number of observations for normality in variables, the results are subject of other approaches like non-parametric tests that can be more accurate than traditional normal tests like t-test. Having more data would be necessary to evaluate the differences across altitude and obtain similar performance that other statistical tests can produce to derive conclusions and insights about viral load and altitude.

## Results

### General Results

4,929 people tested positive for SARS-CoV-2 and from this, 51.3% (*n* = 2530) were men and 48.7% (*n* = 2399) were women. 43% (*n* = 2135) came from the low altitude jurisdictions and 57% (*n* = 2794) came from high altitude locations. In terms of self-reported symptoms, highlanders have a slightly lower proportion of symptomatic patients (51,2%) versus those tested at lower elevations (52,0%).

### Age and sex differences

From the total sample of 4,929 patients, the average age was 38 years (SD = 17.76). The average age for women was 38 years (SD = 18.1) while for men was 38 years (SD = 17.5).

**Figure 2:**
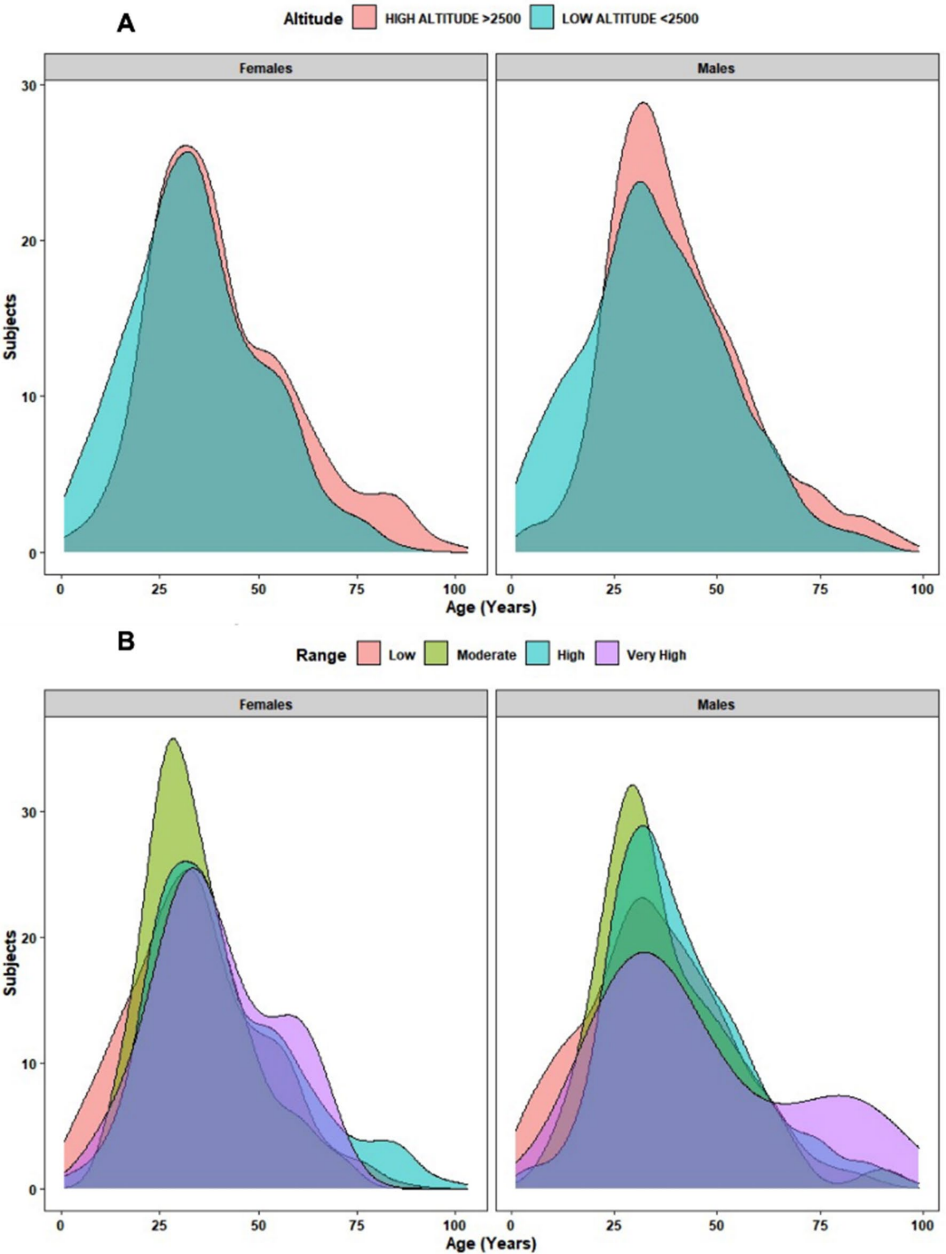
Age and sex distribution per Altitude ranges among SARS-CoV-2 RT-qPCR positive test from 4929 patients. Panel A: Low (< 2500 m) and High altitude classification (> 2500 m). Panel B: Low altitude (< 1500 m), Moderate altitude (1500 to 2500 m), High a.

In terms of age by elevation categories, we found that at low altitude, men have an average age of 35 years (SD = 17.7) while high altitude 40 years (SD = 17.7). On the other hand, low altitude women have an average of 34 years (SD = 16.8) while high altitude 41 years (SD = 18.5), being any of these differences, statistically significant (*p*-value < 0.001 for women across high and low altitude and *p*-value < 0.0001 for men) Fig. 2.

### Viral load analysis

**Figure 3:**
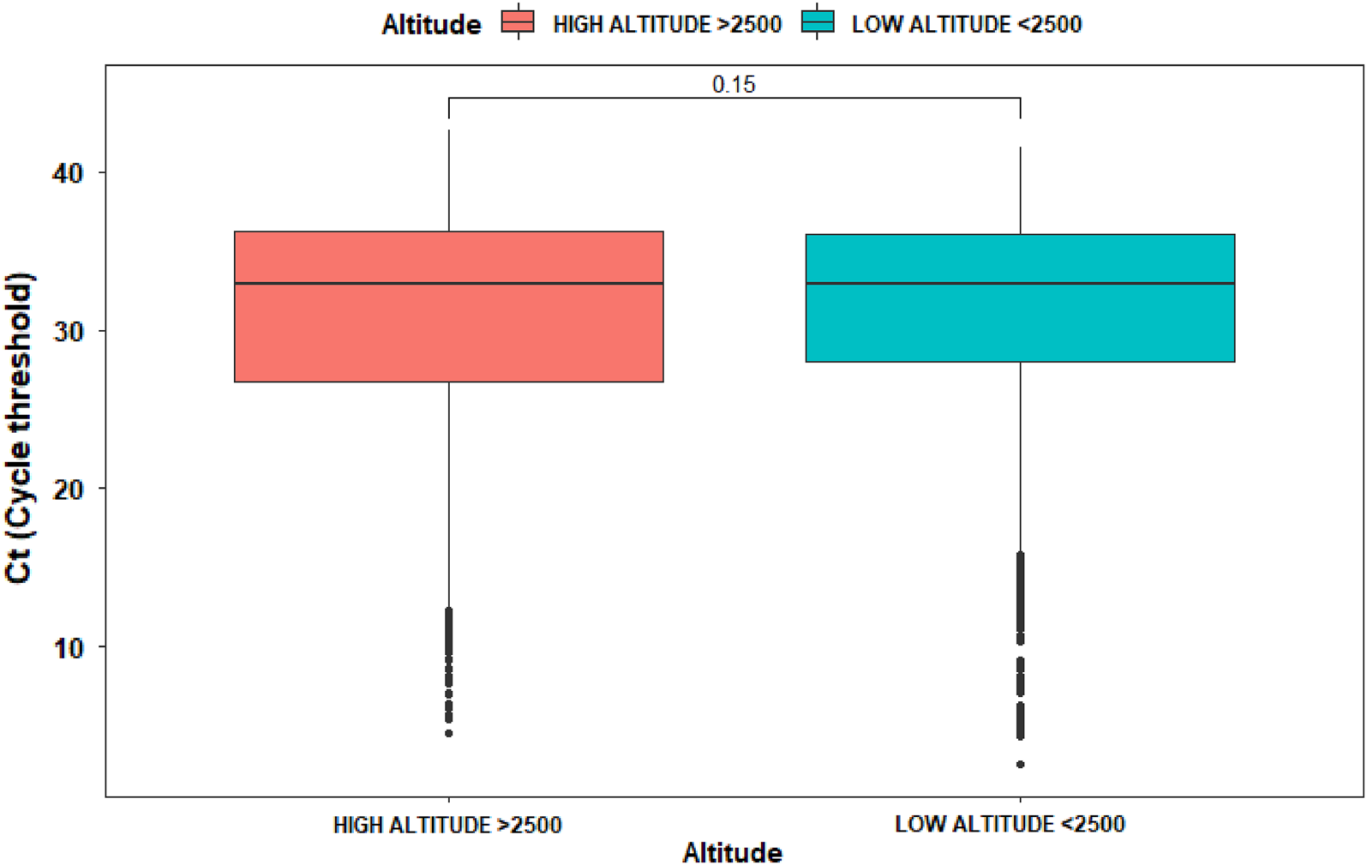
Box plot of viral load composition across altitude and sex of SARS-CoV-2 test positivity among 4929 tested people.

The average viral load at low altitude was 170,054,839,743.98 copies/ml (SD = 7,093,792,305,641.90 copies/ml) and at high altitude was 54,177,678.82 (SD = 1,008,390,937.76 copies/ml), being this difference not significant (p-value of 0.27). When considering sex, women had an average viral load of 143,244,371,554.75 copies/ml (SD = 6,652,794,470,175.70 copies/ml) and men had an average viral load of 6,384,471,198.29 copies/ml (SD = 316,562,541,098.43 copies/ml), being this difference not statistically significant (*p*-value of 0.31) Fig. 3.

**Figure 4:**
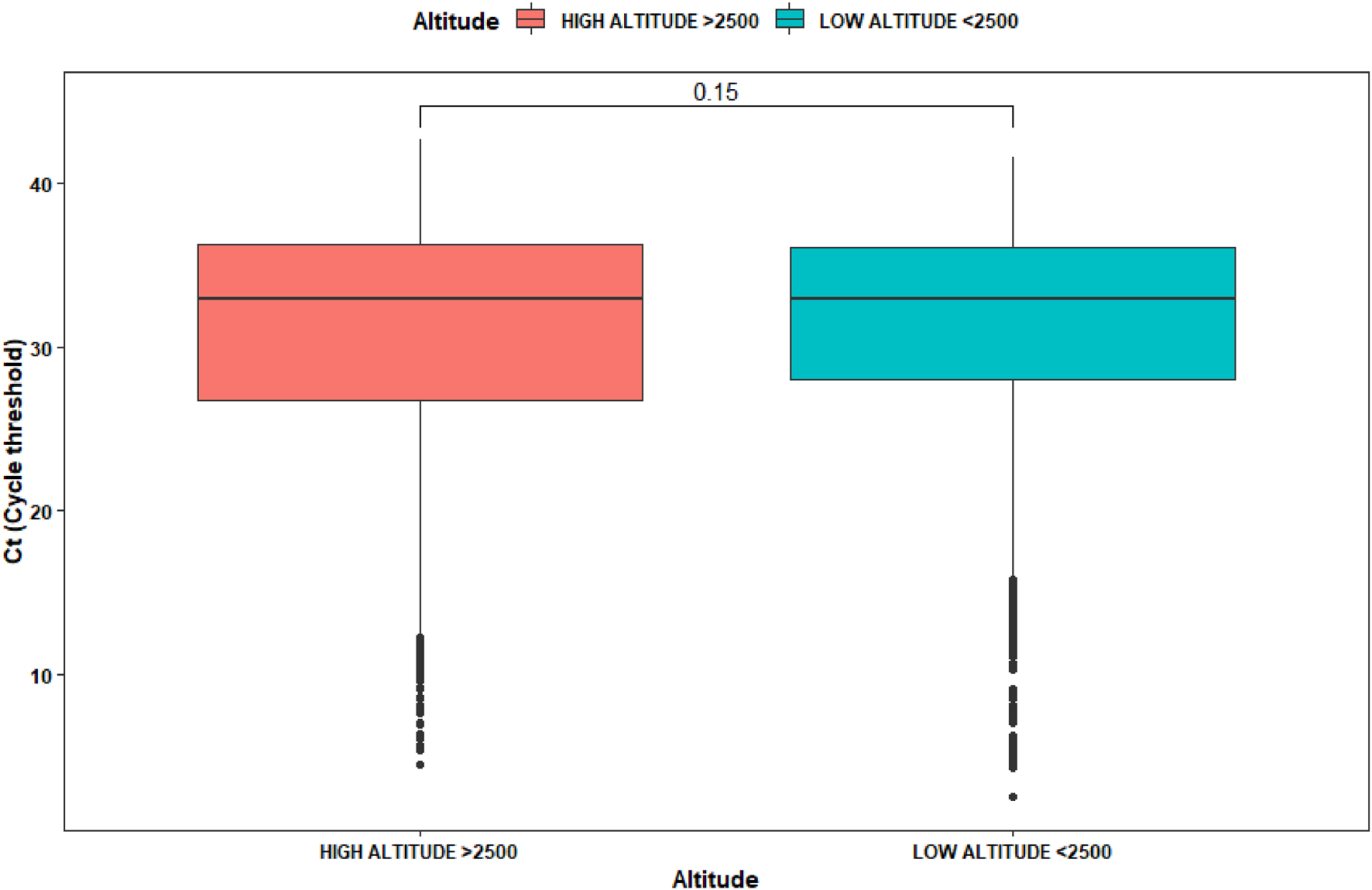
Box plot of viral load (measured as Ct) at low and high altitude due to SARS-CoV-2 infection within 4929 test that resulted positive.

In the case of Ct cycles, we have applied a simple *t*-test to determine possible differences across sex and altitude. For high altitude there is not difference in Ct (*p*-value of 0.95 with women having an average Ct of 30.72 and men a Ct average of 30.75). For low altitude it was possible to determine that Ct values for women was 30.7 and for men 31.3 (Fig. 4).

### Viral load analysis at low, moderate, high, and very high altitudes

**Figure 5:**
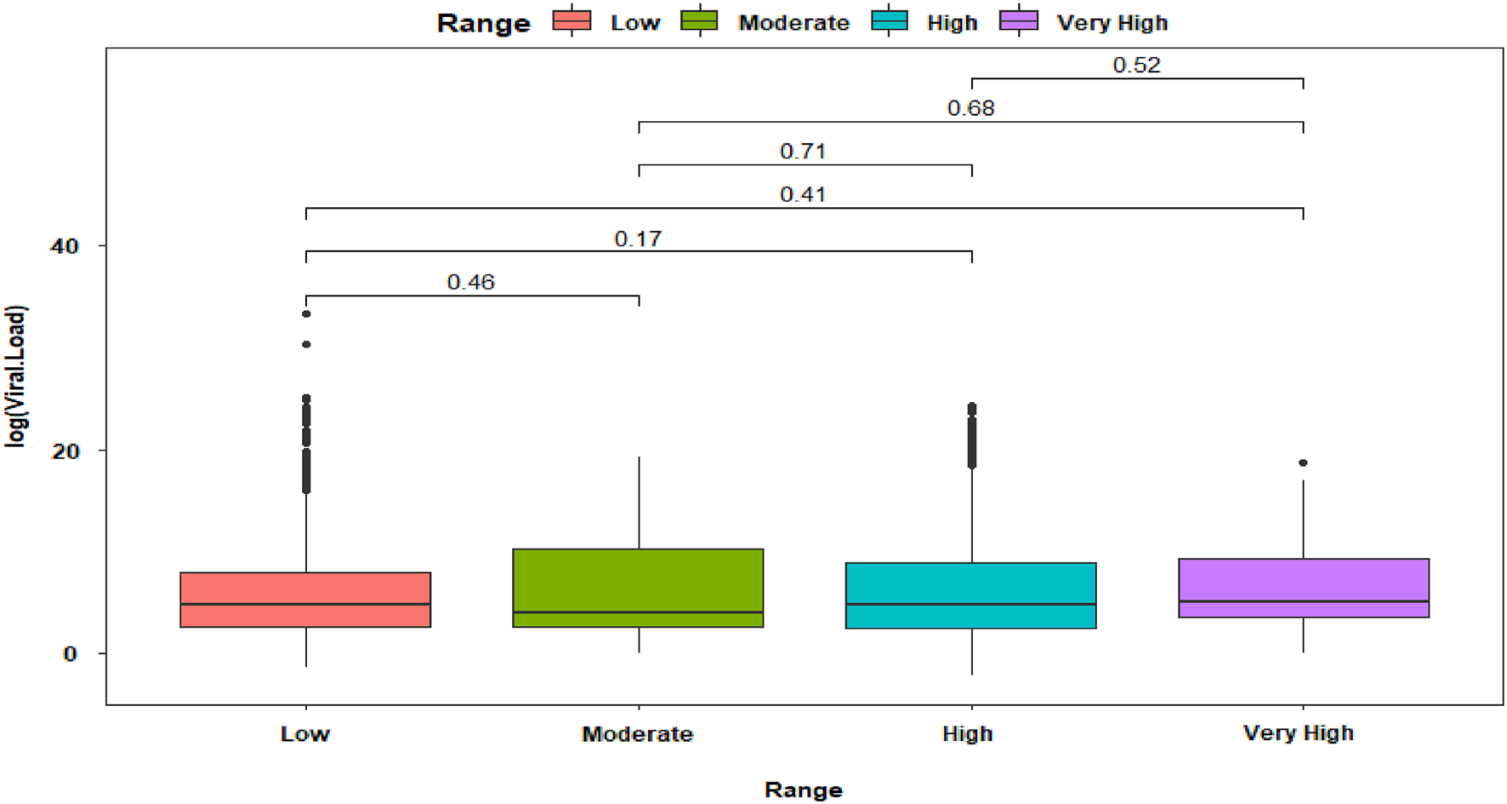
Box plot of viral load composition across altitude range of SARS-CoV-2 test positivity among 4929 tested people.

Viral load average at low altitude was 177,788,219,075.88 (SD = 7,253,286,130,352.97), moderate altitude was 5,476,632.75 (SD = 28,181,259.64), high altitude was 54,620,377.62 (SD = 1,013,118,863.64) and very high altitude 7,200,525.49 (SD = 28,650,823.76) (Fig. 5).

**Figure 6:**
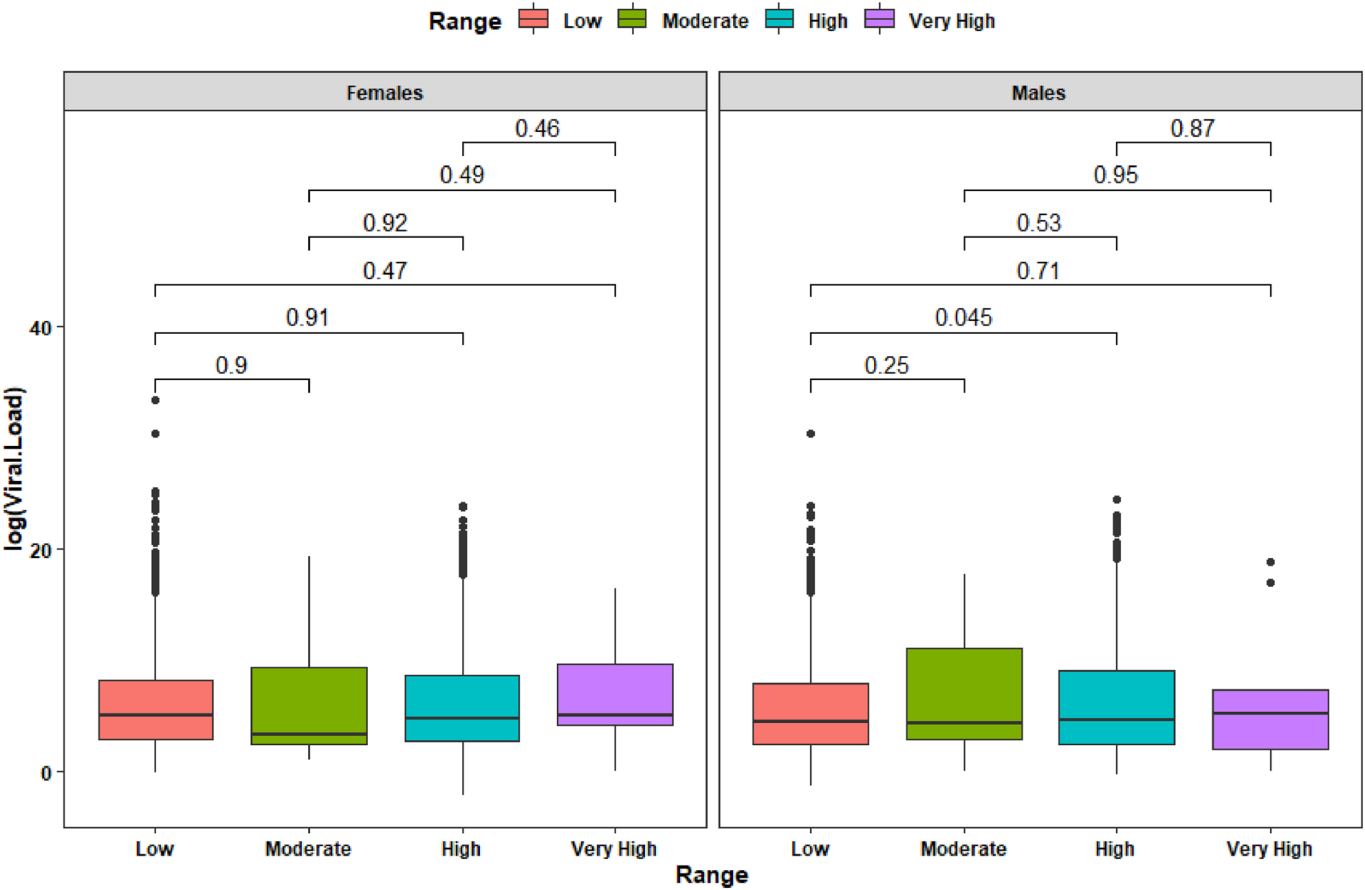
Box plot of viral load composition across altitude range of SARS-CoV-2 test positivity among 4929 tested people by sex.

In the case of women living at low altitude, we found an average viral load of 353,274,233,020.88 copies/ml (SD = 10,448,455,198,449.50 copies/ml), at Moderate altitude 9,818,113.35 copies/ml (SD = 40,128,245.66 copies/ml), at High altitude 50,732,280.82 copies/ml (SD = 870,604,774.50 copies/ml) and at Very High altitude there is an average viral load of 1,238,566.60 copies/ml (SD = 3,612,934.90 copies/ml). In the case of men living at Low altitude there is an average viral load of 15,307,424,643.64 copies/ml (SD = 491,436,337,911.20 copies/ml), in Moderate altitude there is an average viral load of 1,587,389.71 copies/ml (SD = 7,008,152.82 copies/ml), in High altitude there is an average viral load of 58,361,910.31 copies/ml (SD = 1,133,773,394.75 copies/ml) and in Very High altitude there is an average viral load of 15,330,469.43 copies/ml (SD = 43,679,247.00 copies/ml) (Fig. 6).

**Figure 7:**
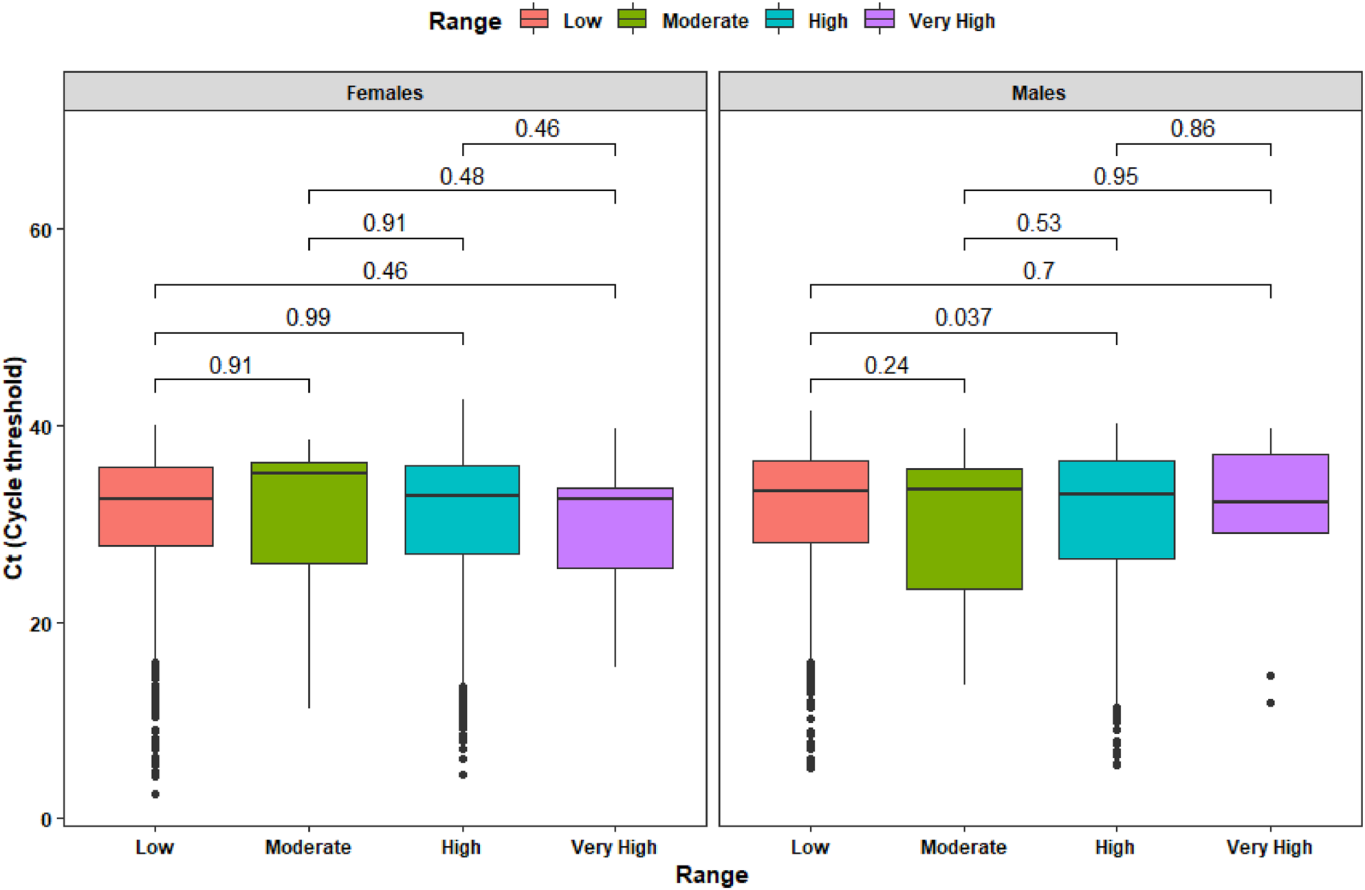
Box plot of Ct composition across altitude range of SARS-CoV-2 test positivity among 4929 positive patients.

When comparing viral load by sex and according to four altitude categories, we could not find statistically significant differences. In terms of Ct, when sex is included as covariate using altitude range, at least one relationship between low and high altitude can influence on Ct (*p*-value 0.037). Despite other combinations show p-values greater than 0.05 displaying same trend as the previous test, the presence of extreme values can be a reason to get such as results, but adding a new variable produce one significative comparison explaining that more variable can affect Ct on altitude range, but the same variable itself cannot be used to state effects over Ct (Fig. 7).

### Viral load analysis by age groups at low, moderate, high and very high altitudes

There were no statistically significant differences between age groups and altitude after conducting t-test using as reference the age group. Most of p-values are greater than 0.10 showing that differences in viral load for altitude levels are not present across age groups. Even using altitude levels for comparison in age groups showed no evidence of any difference in viral load for altitudes. The only difference that can be found is the distribution of viral load for young adults compared to the same group of people living at high altitude (Table 1).

**Table 1:** Viral loads by age and altitude range among patients with a positive infection for SARS-CoV-2.

	Low		Moderate		High		Very High	
	*N*	Ct Copies/ml	*N*	Ct Copies/ml	*N*	Ct Copies/ml	*N*	Ct Copies/ml
Pre school	76	120,728,727	26	104,510	0	0	0	0
School	136	19,489,756	42	763,174	1	895,112	1	154
Adolescent	226	70,732,899,123	152	19,567,249	10	810,846	1	191
Young adult	860	19,098,980,795	1311	72,289,578	50	4,690,685	13	2,841,347
Adult	637	523,847,629,773	946	50,326,524	26	9,179,921	7	706,238
Elderly	109	26,880,399	291	19,231,731	4	4,039,445	4	36,333,035
Total	2044	102,307,768,096	2768	27,047,128	91	3,269,335	26	6,646,827

**Figure 8:**
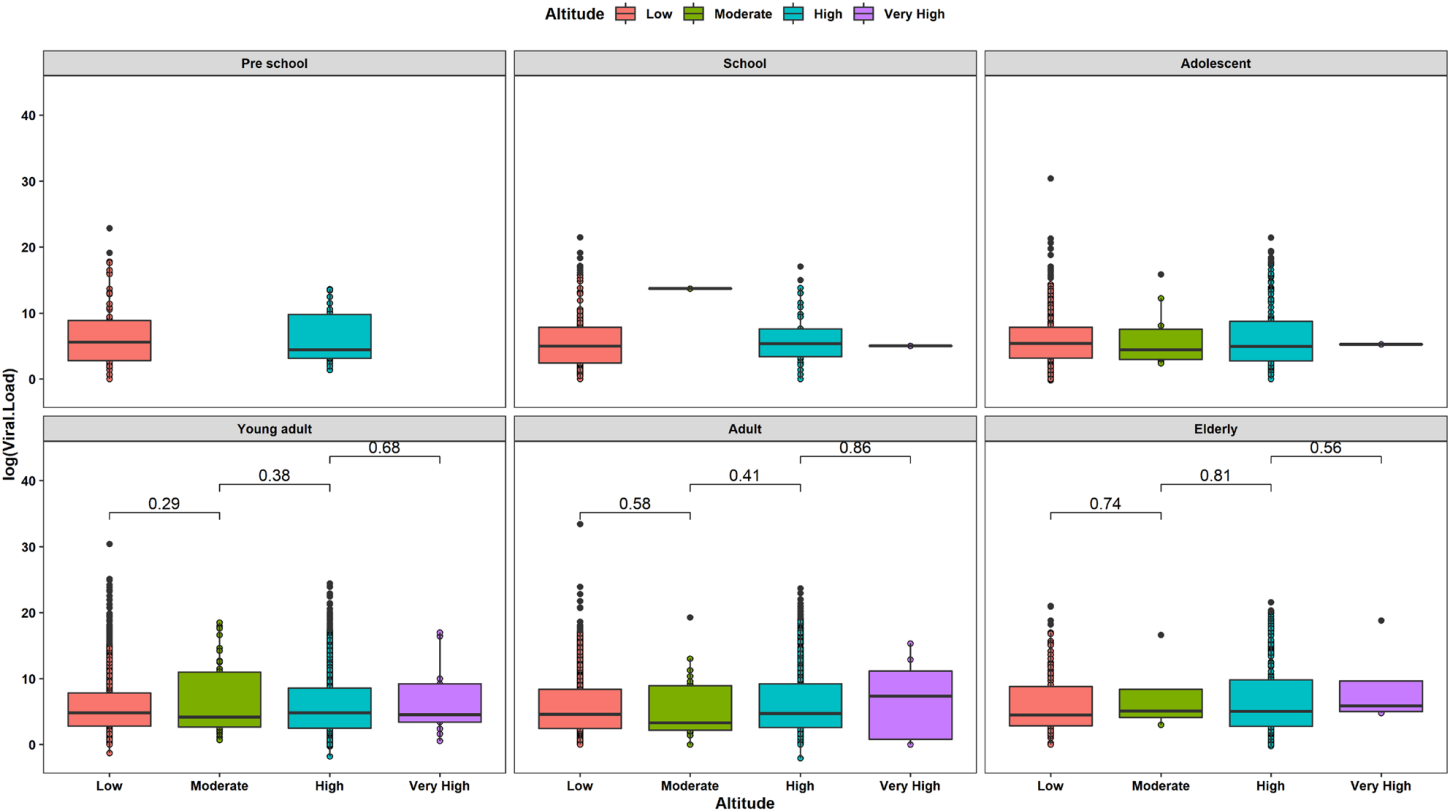
Box plot of viral load composition across age groups and altitude ranges from a sample of 4929 patients with a positive RT-qPCR positive test for SARS-CoV-2 infection.

Despite this, we found that elderly dwellers living at high altitude have higher viral load than the elderly living at low altitude and those living at high altitude (Fig. 8).

### Low, moderate and high viral load using Ct values

**Figure 9:**
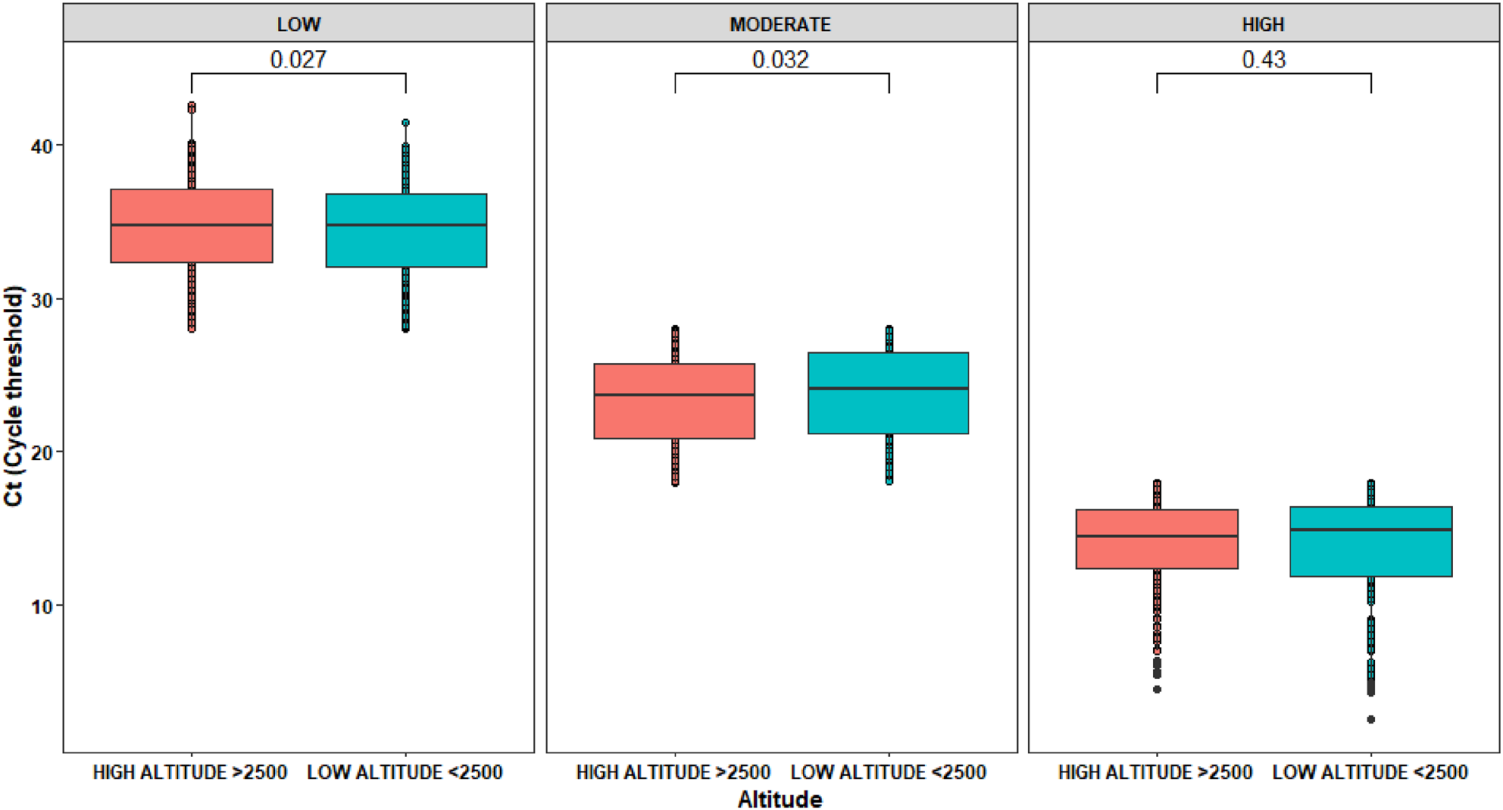
Viral load comparison among elevation groups.

After comparing viral loads by category (low viral load, > 28, moderate viral load, 18–28; and high viral load Ct < 18), we found that low and moderate viral loads do vary at different altitudes; however, at high altitude, these differences were not observed (Fig. 9).

## Discussion

Our exploratory results found no significant difference between people living at high altitude and those living at low altitude locations in terms of SARS-CoV-2 viral load. Our reports are the first one available in terms of exploring this issue at different elevations, and according to our results it seems like living at different altitudes might not play any important role in terms of viral load^28^.

In some investigations, the role that hypobaric hypoxia could have on the viability of the SARS-CoV-2 virus has been analyzed^13,29–31^. In very few reports it is mentioned that ozone could affect viral viability, humidity could affect transmissibility and ultraviolet rays (UV lights) could eliminate the virus faster than in other locations where there is less UV light exposure than at high altitude^32,33^.

There are studies that indicate the influence of environmental factors on the virulence of SARS-CoV-2 at high altitude, among which are dry air, sudden changes in temperature, and ultraviolet radiation levels, as far as this last factor is known. that ultraviolet light A and B is capable of altering the molecular bonds of DNA and RNA in terms of its effect on SARS-CoV-2 it is believed that it could shorten the half-life of the virus^34,35^.

Another factor often linked to the transmission of the SARS-CoV-2 virus may be cold weather. Several studies have suggested that the susceptibility of the virus to temperature may be affected by climate^36–38^. Although cold temperatures have been associated with increased risk to develop some respiratory diseases such as influenza or other respiratory viruses, it is clear that climate has a more important factor influencing people’s behavior^39–41^. For instance, people who live in places where the climate can be adverse tend to socialize less, stay in smaller family or “social pockets”, to remain in-doors more often, thus, have less risk of being part of higher risk transmissibility activities^42–44^.

Besides viral transmission, it seems likely that high altitude may have an important role in improving the survival of some seriously ill patients who live at high altitudes^45^. Even with the presence of comorbidities, survival among severely ill COVID-19 patients at high altitude seems to be improved when compared to low altitude patients, probably linked to their adaptation status that might improves oxygenation^45,46^.

Most of the reports that have described reduced mortality are observational population-based studies rather than individual-based^5,9,16,47,48^. In this sense, on populations but not on individuals. This confers a limitation since important data, such as viral load, have never been measured in high altitude. A recent report by Arias-Reyes et.al 2021, investigated whether the transmission rate of SARS-CoV-2 differs between low and high altitudes^49^. They found that after using a mathematical SEIR model, the probability of viral transmission is lower at high altitude, concluding that their findings strongly support the hypothesis of decreased SARS-CoV-2 virulence in highlands compared to lowlands^49^.

Although the available results suggest lower COVID-19 related mortality at high altitudes, lower viral load cannot be attributed as one causal factor. Viral loads difference among low and high altitude dwellers is unlikely, mainly since the idiopathic response of each organism towards viral replication depend on immunological and biological factors more than in environmental or socio-demographic differences^50–52^.

It is essential to mention that the number of tests performed in different geographic locations may vary, especially in remote rural areas such as those found at high altitudes^53^. For example, in the early stages of the pandemic, the country was ranked 152 out of 178 in terms of tests performed per capita^5^; however, during the later stages of the pandemic, although diagnostic capacity improved, we never reached desirable levels or those recommended by the World Health Organization^54^. In that sense, the positive testing rate (PTR%) was above 5% during most of the outbreaks in Ecuador. For this reason, our viral load testing analysis excluded PTR%, since having an individual result of the amount of virus replicating in each patient confers an exciting approach to the outgoing debate about the impact of high altitude on SARS-CoV-2 replication or virulence.

The question of whether the SARS-CoV-2 viral load among high-altitude dwellers is different from that of low-altitude dwellers remains unresolved, yet our study aimed to dilucidated if any difference in terms of viral load at different altitude ranges exist.

## Limitations

We have identified several limitations of our study. The first and most important is that the viral load was not analyzed in relation to the day of symptom onset, therefore it is not possible to calculate correctly when the peak of transmission was reached in one or another person. Second, it was also not possible to control for factors such as immune status or the use of medications that might reduce viral load or decrease immune response. Finally, causal relationship cannot be established with type of study, but our results may be the door for future, better controlled analyses to try to determine whether SARS-CoV-2 viral loads change with altitude.

## Conclusions

Being the first to analyze viral load at different altitudes, this study allows us to answer one of the last questions about the debate on altitude and its effect on SARS-CoV-2 virulence. Our results support the theory that although adaptation to hypoxia would seem to protect people from more severe COVID-19, the amount of replicating virus at different altitudes is independent of elevation. Our study demonstrates that viral loads (Ct and copies/ml) do not differ from low, moderate or high altitude. Using sex as a co-factor, we found that men have higher viral loads than women at low and moderate altitude locations, while living at high altitude, no differences were found. When Ct values were aggregated by low, moderate, and high viral load, we found no significant differences when sex was excluded from the analysis. We conclude that viral load is not directly affected by altitude, but COVID-19 incidence and mortality are rather affected by socio-demographic and idiosyncratic dynamics.

## Data Availability

The datasets generated analyzed during the current study are available in the following repository: https://github.com/covid19ec/DataCharges.
